# Commencement Day

**Published:** 1894-05

**Authors:** 


COMMENCEMENT DAY.—"In the spring time the young man's mind lightly turns to thoughts of love," says Thompson; but the average medical student crams on Smith's Compend, and prepares for examination. With hesitation, trepidation and perspiration he approaches that green baize door which, veiling his future, conceals a terror in the shape of a bald-headed professor, in whose hands hangs the destiny of many fellows, each, not by a thread—but by a string—of hard questions. Happy they, the happiest of their kind to whom Pat, the janitor, hands a long round tin box next day; while, with a grin, he suggestively protrudes his left hand for the expected fee, never less than a V. Who so proud, then, as they—the fledglings—the new-born medicos?—as when next they meet, the old familiar "Tom" and "Harry" are dropped, and it is "good morning, *doctor;* accept my congrats;—didn't old Blimber make a fellow sweat?"—"Oh shaw, *doctor*, he was *nothing* to old Bones when he got me on the ligaments. I was up to date, tho', you bet; crammed. So long, doctor." (Another two.) "Ah, good morning, doctor; got through, I hear; yes, it was tough. Be on hand to-night, of course, with your swallow tail?" (Exit.)

The palpitating part of it had only *begun*, however, in the green room. (How provokingly old Bones *did* grin when he asked them to "give him the muscles of the neck.") All these young M. D.'s have to stand the battery of bright eyes to-night at the opera house; and in that large and fashionable audience all a-flutter with fans and furbelows—every young fellow has a bright particular pair of eyes that to him look like the rising sun, as he steps out in response to his name to get his sheep skin; while to the owner of said pair of rising-sun orbs, that particular name on the program,—it may even be Grubbs,—blazes with a holy light, quite eclipsing all the others.* Then,—the first time SHE calls Harry "doctor,"—oh, not for the crown of an Indian prince would he exchange that proud title! (We've been there—though it was in the long, long ago; memory brings back the days that are no more,)—and—at the ball; and—after the ball; what medicine—heart excitants mostly, we fear, is talked, as,

---

* (And the band played Annie Laurie.)

arm in arm, each happy couple promenades beneath the vine-clad trellis, or—drop the curtain here; the "sweetness" of that faithful watch dog's honest bark, that Byron tells us about, baying deep-mouthed welcome as, in after years we draw near home any rainy, dark night, after a ten mile ride for a bare "thankee," is just only brown sugar to double-distilled saccharine—compared to the bliss of those moments, spent with Dulcinea the first evening he wore his title and his pigeon-tail coat; as they told and listened to 'neath the umbrageous shades of those grand old oaks, the old, old tale; it is always the same; told with variations often, perhaps, but always the same old tale;—and ever new;—told with the eyes, "for the heart doth speak when the lips move not"—so that when flashed from a woman's eyes even a savage can comprehend: "two souls with but a single thought," etc., etc.; ah, me, would I were a boy again; or, rather, a young doctor, sprouting our first mustache. How much medicine we then *did* know, good gracious! The wonder grew sure enough—with us—that one small head could carry it. But somehow one's head seems to leak medical knowledge, as the bones harden and the sutures close up. Just the reverse we would expect; but it is a fact; we think that most any doctor of our age will admit it;—the older we get the less we know! Crowded out, p'haps, to make room for a recollection of our uncollected bills (or our unpaid ones); or by family cares, and calculations how to make a $2 fee buy Johnny those red-top boots promised him at Christmas; and the dear wife—she of the sun-rise eyes of long ago—that pretty calico dress she saw in Isaac's window!

Ah, yes; spring time is "commencement" time; and the output of the new issue of (we like to have said "greenbacks," so absorbed are we in studying out the above financial sphynx—no, the silver question and the Bland bill)—the output of the new generation of doctors is large. We shall not attempt even to keep a memorandum of the number; each college is turning them out by the score,—raw material that, beyond doubt, will make the future Sir Andrew Clark, the S. W. Gross, the Austin Flint, the Marion Sims.

To them all, those who are properly imbued with the love of science; who have chosen medicine not as a money getter alone—we say *aim high!* What was possible to the poor Southern boy Sims, Wyeth, Nott, is possible to you;—do not put away your books now that you have your diploma; you have only *graduated*, you have not *finished;* you have only begun. It is truly your

"commencement day." Drink deep, or touch not the Pyærian Spring. Let not alone the sun-rise eyes of your beloved inspire you; determine to win a place for her where, in after years, she may not be ashamed of her young doctor. The hill whereon fame's proud temple shines afar *is* hard to climb; but it has been climbed. What others can do, you can do; so my dear boy (*beg* pardon)—my *dear* young doctor—aim high,—and *Amen.*

